# Incidence, deaths, and lifetime costs of injury among American Indians and Alaska Natives

**DOI:** 10.1186/s40621-019-0221-z

**Published:** 2019-11-11

**Authors:** Amanda A. Honeycutt, Olga Khavjou, Simon J. Neuwahl, Grant A. King, Meredith Anderson, Andrea Lorden, Michael Reed

**Affiliations:** 1 RTI International, Public Health Economics Program, 3040 Cornwallis Road, Durham, NC 27709 USA; 2 Kauffman & Associates, Inc, Washington, DC USA; 3 Independent Health Services Research Consultant and Department of Health Administration and Policy, University of Oklahoma Health Sciences Center, Oklahoma City, OK USA; 4 Indian Health Service, Division of Environmental Health Services, Rockville, MD USA

**Keywords:** American Indian, Alaska native, Indian Health Service, injury, Costs, Prevention

## Abstract

**Background:**

In the United States, the mortality burden of injury is higher among American Indians and Alaska Natives (AI/AN) than any other racial/ethnic group, and injury contributes to considerable medical costs, years of potential life lost (YPLL), and productivity loss among AI/AN.

This study assessed the economic burden of injuries for AI/AN who are eligible for services through Indian Health Service, analyzing direct medical costs of injury for Indian Health Service’s users and years of potential life lost (YPLL) and the value of productivity losses from injury deaths for AI/AN in the Indian Health Service population.

**Methods:**

Injury-related lifetime medical costs were estimated for Indian Health Service users with medically treated injuries using data from the 2011–2015 National Data Warehouse. Productivity costs and YPLL were estimated using data on injury-related deaths among AI/AN in Indian Health Service’s 2008–2010 service population. Costs were reported in 2017 U.S. dollars.

**Results:**

The total estimated costs of injuries per year, including injuries among Indian Health Service users and productivity losses from injury-related deaths, were estimated at $4.5 billion. Lifetime medical costs to treat annual injuries among Indian Health Service users were estimated at $549 million, with the largest share ($131 million) going toward falls, the most frequent injury cause. Total estimated YPLL from AI/AN injury deaths in Indian Health Service’s service population were 106,400. YPLL from injury deaths for men (74,000) were 2.2 times YPLL for women (33,000). Productivity losses from all injury-related deaths were $3.9 billion per year. The highest combined lifetime medical and mortality costs were for motor vehicle/traffic injuries, with an estimated cost of $1.6 billion per year.

**Conclusions:**

Findings suggest that targeted injury prevention efforts by Indian Health Service likely contributed to lower rates of injury among AI/AN, particularly for motor vehicle/traffic injuries. However, because of remaining disparities in injury-related outcomes between AI/AN and all races in the United States, Indian Health Service should continue to monitor changes in injury incidence and costs over time, evaluate the impacts of previous injury prevention investments on current incidence and costs, and identify additional injury prevention investment needs.

## Background

Injuries among American Indians and Alaska Natives (AI/AN) in the United States cause high medical costs, years of potential life lost (YPLL), and productivity losses (Indian Health Service. Indian health focus, 2017). Unintentional injuries are the leading cause of death for AI/AN ages 1 to 44 and the third-leading cause for all ages of AI/AN, with an age-adjusted mortality rate in 2009 of 91.9 per 100,000, nearly 2.5 times the rate among all races (37.5) and 2.3 times the rate for U.S. Whites (39.5) (Indian Health Service. Indian health focus, 2017). Suicide, an intentional injury, is the eighth-leading cause of death among AI/AN, with a mortality rate of 19.6 per 100,000, 66% higher than the rate for all U.S. races (11.8) (Indian Health Service. Indian health focus, 2017). The rate of unintentional injury deaths among AI/AN was 111.8 per 100,000 for 2008–2010, accounting for 15% of all AI/AN deaths (Indian Health Service. Indian health focus, 2017). Only death rates from cancers (116.7 per 100,000) and heart disease (115.8 per 100,000) exceeded the rates of unintentional injury deaths among AI/AN from 2008–2010 (Indian Health Service. Indian health focus, 2017). A more recent Centers for Disease Control and Prevention (CDC) report indicates this trend has remained consistent through 2016 (Heron M. Deaths: Leading causes for 2016. National vital statistics reports [Internet], 2018).

Non-fatal injuries also have higher incidence rates and costs among AI/AN than among people of other races and ethnicities, especially for young adults and children (Chikani et al., 2016; Rutland-Brown et al., 2005). The incidence rate of unintentional injuries among AI/AN children was 1.2 to 2.3 times higher than the incidence among White children, while the mortality rate was 1.8 to 8.2 times higher for AI/AN children (Möller et al., 2015). Treating injuries among AI/AN is costly for Indian Health Service (IHS), with 14% of the total medical expenses paid by IHS for purchased/referred care (PRC) in 2016 going toward injuries (Indian Health Service. Indian health focus, 2017).

The U.S. federal government has a government-to-government relationship with AI/AN tribes that includes a federal trust obligation that assures AI/AN rights to health services. IHS is the primary federal health care provider for AI/AN. IHS serves the health care needs of AI/AN through the provision of direct health care services, funding for tribally contracted and operated health programs, and grants and contracts to urban Indian health programs. This system of health services delivered through IHS-operated facilities, tribally operated facilities, and urban Indian health programs is known as the I/T/U system (U.S. Department of Health and Human Services, 2019).

This study estimated the economic burden of fatal injuries for AI/AN in IHS service areas and the burden of medically treated injuries for AI/AN IHS users, which are a subset of AI/AN in IHS service areas. The analysis generated estimates of lifetime medical and productivity costs for a 1-year snapshot of AI/AN with injuries or injury deaths. IHS can use these estimates to monitor changes over time in total injury costs, to track spending on injuries, to identify injury categories that may need additional investments, and to examine the economic impact of IHS injury prevention programs. Health policy makers and researchers at national, regional, and tribal levels may also use these findings to identify trends and prioritize programs to best address the underlying causes of injury among AI/AN. Additionally, the findings may be useful to injury prevention specialists working in tribal communities to inform their decisions about program priorities.

## Methods

An incidence-based cost-of-illness approach was used to estimate the lifetime economic cost of injuries occurring over 1 year among AI/AN in IHS service areas (Segel, 2006; Honeycutt et al., 2003). A societal perspective was used to estimate the annual incidence and lifetime medical costs of treated injuries. YPLL and productivity losses were estimated for injury deaths. Incidence and costs were estimated by injury cause and by intent. Medical costs were inflated to 2017 U.S. dollars (USD) using the Personal Consumption Expenditures for Health Index (Dunn et al., 2016). Productivity costs were inflated to 2017 USD using a gross domestic product price index (U.S. Bureau of Economic Analysis. National Accounts, Section 1, 2018). Present value calculations used a 3% discount rate (Lipscomb et al., 1996).

A focus was on estimating injury costs for the IHS service population, which consists of all AI/AN who reside in geographic areas within IHS service areas, including counties on and near federal Indian reservations (Indian Health Service. Indian health focus, 2017). About 56% of AI/AN in the United States, or about 2.3 million AI/AN, live in an IHS service area (Indian Health Service. Indian health focus, 2017). Data on injury deaths among AI/AN in the IHS service population were used to estimate YPLL and mortality-related productivity losses by injury cause and intent.

Data on injury-related healthcare use were only available for AI/AN who received healthcare services through IHS. These IHS users, totaling approximately 1.6 million people, are a subset of the full IHS service population. The study team used IHS user population data to estimate injury incidence and direct medical costs by cause and by intent based on external cause of injury codes reported in IHS administrative records. The IHS user population includes patients who had at least one direct (i.e., provided by an IHS-operated, or tribally contracted or compacted facility) or PRC inpatient stay, ambulatory medical visit, or dental visit during the last 3 years. PRC is provided by a non-IHS-operated facility or provider through contracts with IHS.

### Incidence

Injury incidence was estimated for IHS users based on data from fiscal years 2011–2015 on patient care^1^ derived from the IHS National Data Warehouse (NDW) reporting system. The NDW is a state-of-the-art, enterprise-wide data warehouse environment for the IHS national data repository and is managed by the National Patient Information Reporting System team in collaboration with the IHS Division of Program Statistics. The NDW contains only IHS-operated facilities, tribal direct, and contract facilities’ inpatient and outpatient data pertaining to various patient characteristics (e.g., age, sex, principal diagnoses, other diagnoses, community of residence, etc.). The data are collected through the medical records systems at each facility, such as the Resource and Patient Management System. Data include one record per inpatient discharge or outpatient visit and are transmitted electronically to the NDW. Patients who receive direct or contracted health services from IHS or tribally operated programs are registered in the NDW. Registered AI/AN patients who had at least one inpatient stay, ambulatory medical visit, or dental visit during the last 3 years were defined as IHS users. Comparable data were not available for AI/AN in the IHS service population who had not used IHS services in the last 3 years.

Consistent with CDC guidance, methods to identify injuries used the first-listed valid external cause of injury code (Fingerhut, 2010). Injury incidence was estimated overall and by care setting to differentiate the injury severity and allow for comparison with the national incidence of injuries. The following mutually exclusive care settings were used: doctor’s offices, emergency departments,^2^ outpatient settings, transfers,^3^ hospitalizations,^4^ and deaths. Injury cause categories were assigned according to the National Center for Injury Prevention and Control’s (NCIPC) recommended codes and categories (Centers for Disease Control and Prevention, 2018) (See Additional file 1). Incidence was estimated by cause, intent, healthcare setting, age group, and sex. All categories of intent were used in analysis, including unintentional, self-inflicted, assault, other, and undetermined. Intentional injuries were identified as those with intents of either self-inflicted or assault. Injury incidence per 100,000 person years was calculated by dividing the total number of injury cases per stratum for the 5-year data period by total person years per stratum in the IHS user population and then multiplying by 100,000.

### Deaths and years of potential life lost

Injury deaths were estimated for the IHS service population by multiplying age- and sex-specific injury-related death rates from 2008–2010 by the 2008–2010 IHS service population by age and sex. The IHS Injury Report provides estimates of death rates based on AI/AN vital statistics, which are adjusted for misreporting of AI/AN race (Indian Health Service. Indian health focus, 2017). IHS Division of Program Statistics provided 2008–2010 service population data and unpublished rates for motor vehicle and pedestrian-related motor vehicle deaths, because they were not available in the IHS Injury Report. The study team calculated YPLL by multiplying the annualized age- and sex-specific number of deaths for each injury category by the remaining age- and sex-specific life years, assuming a life expectancy of 75 years, consistent with published estimates of injury mortality (Borse N, Rudd R, Dellinger A, Sleet D. Years of potential life lost from unintentional child and adolescent injuries—United States, 2000–2009. J Safety Res [Internet]. 2013 [cited, 2018; Centers for Disease Control and Prevention. Years of potential life lost from unintentional injuries among persons aged 0–19 years—United States, 2000-2009. MMWR [Internet], 2012). YPLL calculations assumed the age of death equaled the mid-point of the age interval. Total YPLL were calculated as the sum of YPLL across all age and sex groups.

The injury death categories reported in the IHS Injury Report are not mutually exclusive because of limitations of death certificate data. Additionally, the IHS Injury Report does not report death rates for each individual injury death cause. This limited reporting made it challenging to directly line up injury deaths by cause and by intent among the IHS service population with estimates of injury incidence by cause and by intent for the IHS user population. However, to generate comparable estimates of costs by intent across the user and service populations, the study team estimated injury deaths and YPLL by intent for unintentional injuries and the two intentional injury categories of homicide and suicide.

### Lifetime medical costs

To estimate the lifetime medical costs of injuries occurring over the 5-year period from 2011–2015, the study team assigned previously published lifetime medical costs of injury to their estimates of injuries among 2011–2015 IHS users (Lawrence & Miller, 2014; Finkelstein et al., 2006). Costs to treat an incident injury include emergency medical transport, emergency department, hospitalization, and/or office-based visit costs. Lifetime costs also include the present value of medical costs in the months and years after the injury. Injury cost estimates were obtained from CDC’s Web-based Injury Statistics Query and Reporting System (WISQARS) cost of injury module for injury deaths and injuries treated in U.S. hospitals and emergency departments among all races (Lawrence & Miller, 2014) and from Finkelstein, Corso, and Miller (Finkelstein et al., 2006) for injuries among all races treated in other settings. Both sources estimated lifetime costs of an incident injury by cause, setting, and sex. Estimates reflect the cost of the incident injury plus subsequent costs for up to 7 years (See Additional file 2). For each incident injury from the 2011–2015 NDW data, costs (Lawrence & Miller, 2014; Finkelstein et al., 2006) were applied by cause, setting, and sex. Average lifetime medical costs per injury event were estimated as total costs by cause and setting from 2011–2015 divided by the total number of incident injuries in the IHS user population by cause and setting from 2011–2015.

### Mortality costs

The study team assigned productivity losses to injury-related YPLL for AI/AN in the IHS service population to estimate injury-related mortality costs by age and sex using a human capital approach where productivity losses were valued using wages as a proxy for employee output (Lensberg et al., 2013). Mortality costs included forgone labor earnings and household production losses due to premature death from injury. The present value of future earnings and household production was calculated using national annual earnings estimates and the household production dollar value (U.S. Census Bureau. Current Population Survey Table Creator [Internet]. Washington, DC: U.S. Census Bureau [cited, 2018; Data, 2014). To avoid overestimating forgone earnings due to injury deaths, average earnings were multiplied by national employment rates, and household production losses were multiplied by the percentage of people who live in households (Haddix et al., 2003). Earnings and household production estimates were adjusted for expected productivity growth using a 1% annual growth rate (Haddix et al., 2003).

Mortality costs were calculated as the present value of lifetime earnings and household productivity losses multiplied by the number of age- and sex-specific deaths from each injury category. Labor and household productivity costs were summed to calculate total productivity losses resulting from injury-related premature mortality.

## Results

Results are first presented for injuries occurring in the 2011–2015 IHS user population. Injury incidence and lifetime medical costs for injuries in the 2011–2015 IHS user data are discussed. Next, results are provided for injury deaths in the 2008–2010 IHS service population. Injury deaths by category are described and the associated lifetime productivity losses and YPLL are presented. Finally, we provide estimates of the total lifetime medical and productivity costs for injuries and injury deaths estimated to occur over a 1-year period among IHS users or the service population. These total cost estimates combine results from analyses of the 2011–2015 IHS user data and the 2008–2010 IHS service population injury death estimates.

### Incidence and lifetime medical costs of injury in the 2011–2015 IHS user population

**Fig. 1:**
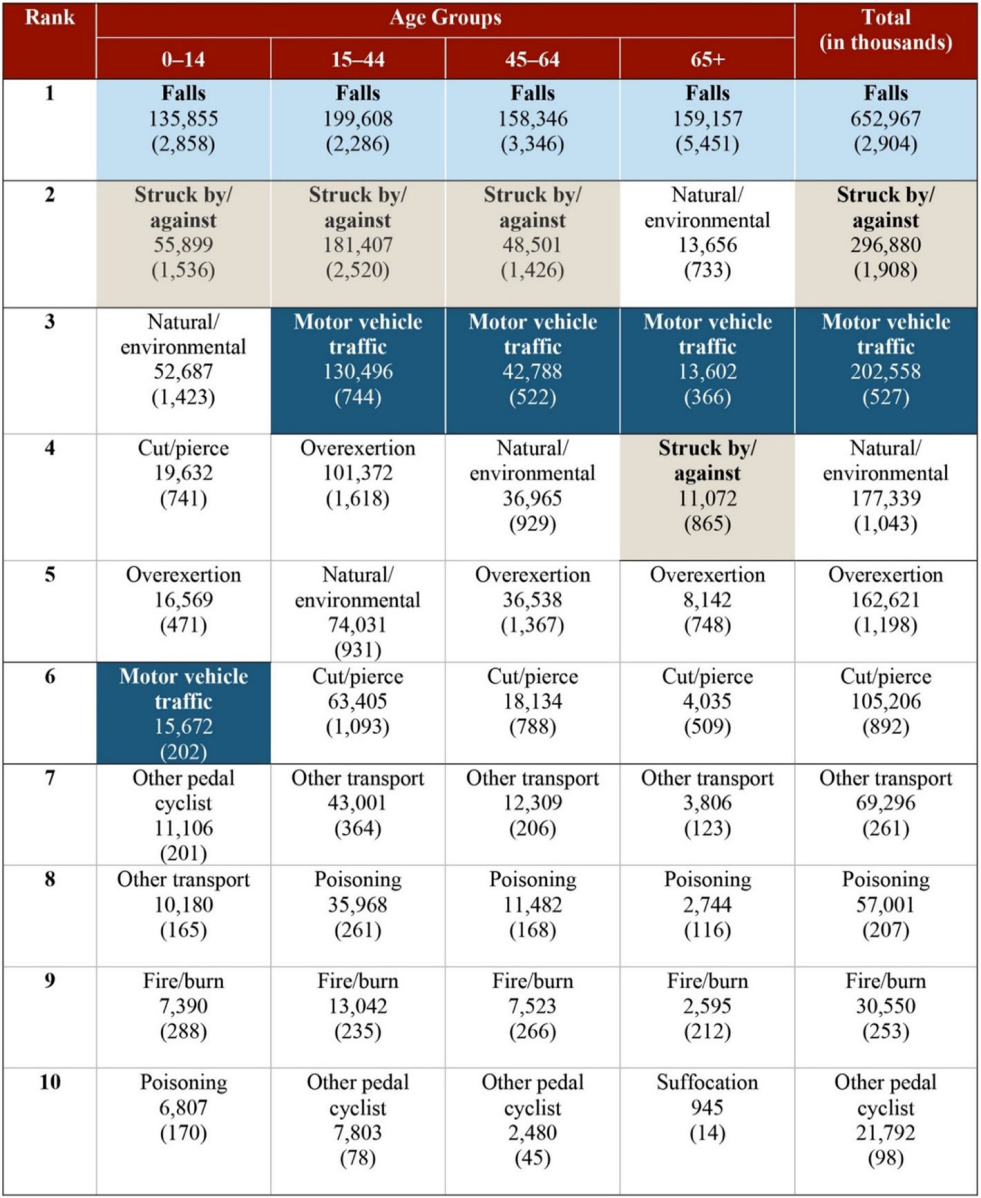
Injury lifetime medical cost rankings among IHS users, by age group, 2011–2015*. Costs are shown in 2017 USD thousands. Incidence rates per 100,000 person-years in the user population are shown in parentheses beneath costs. *Note: Fig. 1 shows the 3 highest cost categories in shaded cells and the 10 highest cost injury categories overall. The three highest categories are shaded in the “total” column; light blue shading indicates a cost ranking of 1, gray shading indicates a cost ranking of 2, and dark blue shading indicates a cost ranking of 3. In the age group columns, the same shading from the total column is used to highlight these three causes and to show how their cost ranking differs by age group. Not shown are suffocation ($9,894,000), machinery ($5,359,000), firearm ($5,061,000), other pedestrian ($3,721,000), and drowning ($724,000)

In the IHS user population, 995,823 non-fatal incident injuries were identified for 2011–2015, representing an incidence rate of 12,202 per 100,000 person years (Table 1). Lifetime medical costs of fatal and non-fatal injuries were estimated at $2.7 billion for this 5-year period of incident injuries. Unintentional injuries accounted for 86.2% (858,929) of the total injuries and $1.8 billion in lifetime medical costs. The rate of intentional injury was 1179 per 100,000 person years, with associated lifetime medical costs of $236.3 million for intentional injuries that occurred in 2011–2015. Within the intentional injury category, 12% were self-inflicted and 88% were from assault. The most frequent injury causes were falls, being struck by or against objects (BS/AO), and overexertion. The costliest per-event injuries were drowning, suffocation, motor vehicle/traffic, other pedestrian injuries, and unspecified injuries. Unspecified injuries had no cause reported, such as fractures with cause unspecified. Falls were the costliest injury among all age groups in the IHS user population, with $653 million in lifetime medical costs for injuries occurring in 2011–2015 (Fig. 1), followed by BS/AO ($297 million) and motor vehicle/traffic injuries ($203 million). The ranking of injuries by incidence matched the ranking by lifetime medical costs with some exceptions. For example, overexertion had lower costs but occurred more often than natural/environmental injuries.

**Table 1:** IHS user injuries over 5 years and lifetime medical costs by intent and cause, 2011–2015*

Injury Category (non-fatal and fatal)	Incidence		Lifetime Medical Cost (2017 USD thousands)	Number with Non-missing Cost Data	Per- Injury Event Cost (2017 USD)
	Count	Rate per 100,000 person years			
All injuries	995,823	12,202	2,743,980	914,014	3000
Injury intent					
Unintentional	858,929	10,525	1,811,158	793,425	2280
Intentional^1^	96,265	1179	236,258	87,986	2456
Other/undetermined	40,629	498	696,564	32,603	17,157
Injury cause					
Falls	237,057	2904	652,967	237,057	2760
Struck by/against	155,654	1908	296,880	155,654	1910
Overexertion	97,772	1198	162,621	97,772	1660
Natural/environmental	85,122	1043	177,339	85,122	2080
Cut/pierce	72,816	892	105,206	72,816	1450
Motor vehicle/traffic^2,3^	43,064	527	202,558	42,292	4790
Other, transportation^2^	21,296	261	69,296	21,296	3250
Fire/burn	20,667	253	30,550	20,667	1480
Poisoning	16,859	207	57,001	16,859	3380
Other, pedal cyclists^2^	8014	98	21,792	8014	2720
Machinery	3315	41	5359	3315	1620
Suffocation	1863	22	9894	1863	5310
Firearms^3^	1387	17	5061	1278	3960
Other, pedestrian^2,3^	1249	15	3721	795	4680
Drowning^3^	221	3	724	102	7100
Other specified^4^	93,075	1141	142,126	93,075	1530
Unspecified^4,5^	136,392	1671	800,885	56,037	14,290

*Note: Injury counts, lifetime medical costs, and per-incident injury costs were calculated including the 349 fatal injuries reported in the NDW injury data. Fatal injuries were not included in incident injury rates to allow for comparisons with non-fatal injury surveillance data from other sources. Per-injury event costs were calculated as total cost by intent/cause divided by the total number of injury events for which costs could be assigned

1. The two categories of intentional injuries are self-inflicted and assault. Twelve percent of intentional injuries were self-inflicted, and 88% were from assault.

2.These are mutually exclusive categories. “Motor vehicle/traffic” crashes include all traffic collisions involving a motor vehicle and pedestrians, cyclists, motorcyclist, or other motor vehicles. “Other, transportation” are train, aircraft, watercraft, off-road, and other non-traffic crashes (not involving a pedestrian or cyclist). “Other, pedal cyclists” are train and non-traffic crashes involving pedal cyclists. “Other, pedestrian” are train and non-traffic crashes involving pedestrians.

3. Cost data were not available for office-based events or outpatient events for these injury categories. The difference between the injury event “Count” and the “Number with Non-missing Cost Data” is the number of events with missing cost data.

4. The “other specified” category includes “other specified and classifiable” and “other specified, not elsewhere classifiable.” Other specified injuries include those with causes not routinely reported (e.g., explosions, electric current, late effects of unintentional injury) or for which injury codes do not exist (e.g., assault by other specified means). Unspecified injuries had no cause reported, such as fractures with cause unspecified. Additional detail on missing NDW data and the impact on injury counts, rates, and costs is also provided in Additional file 1.

5. Unspecified injury events did not have cost data for office-based events or outpatient events. More than half of all “Unspecified” injury events were missing cost data. Because of the high cost of “Unspecified” hospital and emergency room visits (Additional file 2 Table B1), per-injury event costs for “Unspecified” injuries are very high ($14,290).

### Deaths, YPLL, and productivity costs resulting from injury in the 2008–2010 IHS Service population

**Fig. 2:**
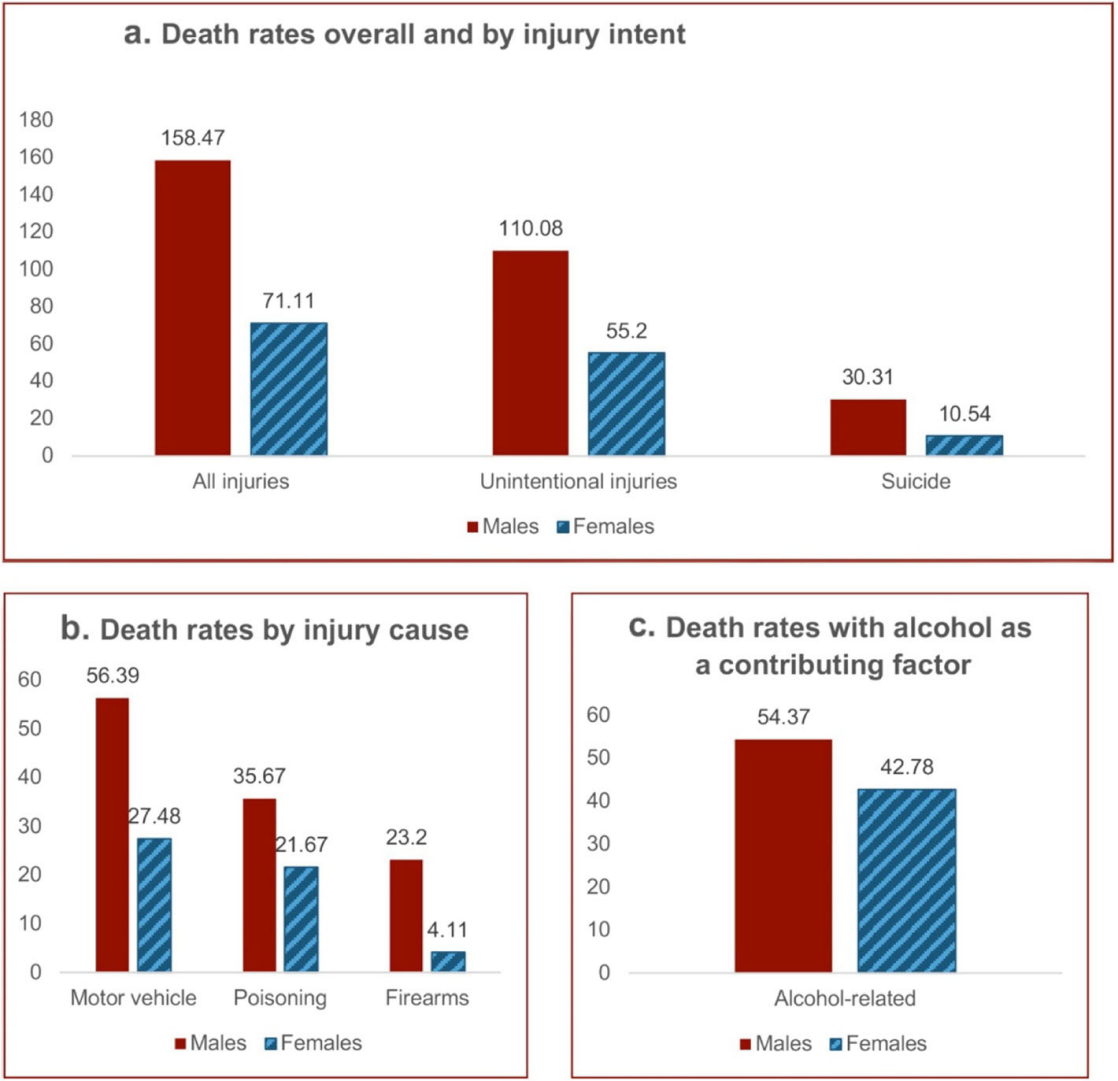
**a**. Injury death rates among IHS AI/AN service population overall and by injury intent*. **b**. Injury death rates among IHS AI/AN service population by injury cause*.**c**. Injury death rates with alcohol as a contributor among IHS AI/AN service population*. * **a**, **b**, and **c** show 2008–2010 injury death rates per 100,000-person years. Not shown are injuries with death rates lower than 20 per 100,000 in males and females, including intent of homicide (18.1 male, 5.37 female) and causes, including pedestrian-related motor vehicle (11.5 male, 3.94 female), falls (6.92 male, 4.08 female), drowning (5.12 male, 0.98 female), and fire and smoke (2.62 male, 1.81 female)

Total injury-related death rates in the IHS service population were 115 per 100,000, with the rate more than twice as high among men (158) than among women (71) (Fig. 2). Most of the deaths were from unintentional injuries. For injury death causes alone, motor vehicle/traffic injuries had the highest death rates, followed by poisonings (Fig. 2b). Alcohol contributed to injury deaths across all causes and intents (Fig. 2c), and alcohol-related injury death rates were 54.4 per 100,000 person years for men and 42.8 per 100,000 person years for women. Although alcohol is a contributing factor in these deaths and not an underlying cause, it is an important factor to consider in planning for injury prevention.

**Fig. 3:**
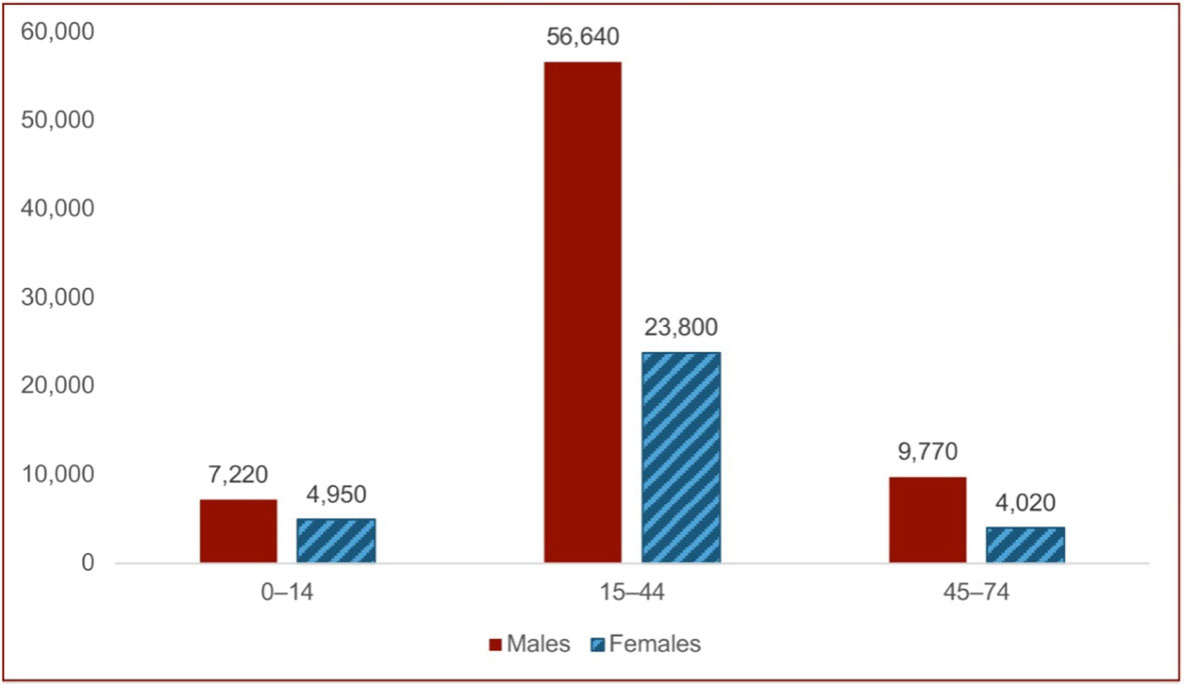
Annual injury death YPLL* by age group (2009–2010 IHS AI/AN service population). *calculated YPLL by assuming a life expectancy of 75 years for injury deaths estimated to occur during 1 year

An estimated 106,400 YPLL were lost annually due to injury among AI/AN in the IHS service population. Men experienced more than twice as many YPLL (73,630) than women (32,770) (Fig. 3). Adults ages 15–44 experienced the majority of YPLL (76%). Costs from productivity losses due to annual injury deaths in the IHS service population were estimated at $3.9 billion, with an estimated total cost of $1.5 million per injury death (Table 2). Unintentional injuries accounted for nearly $2.7 billion of productivity costs. Injury deaths related to alcohol use and abuse contributed to $1.1 billion of productivity costs across all causes and intents. Mortality costs of $1.6 billion were attributed to motor vehicle/traffic injuries; the next highest cause of mortality costs was poisonings at $949 million.

**Table 2:** Estimated annual AI/AN premature mortality costs by injury (2008–2010 IHS service population)

Injury Category	Estimated Annual Number of Deaths	Productivity Costs (2017 USD millions)			
		Labor	Household Productivity	Total (Labor + Household Productivity)	Total Cost per Injury Death
All injuries^a^	2657	3175	727	3902	1.47
Unintentional injuries	1914	2151	509	2660	1.39
Suicide	473	646	140	786	1.66
Homicide	271	378	78	456	1.68
Injuries by cause					
Motor vehicle	971	1285	297	1582	1.63
Pedestrian-related motor vehicle	179	233	51	284	1.59
Firearms	316	453	87	541	1.71
Poisoning	664	760	189	949	1.43
Falls	127	64	15	78	0.62
Drowning	71	92	18	110	1.55
Fire and smoke	51	55	14	69	1.36
Alcohol-related injuries	991	887	233	1119	1.13

^a^**Note:** The “All injuries” category was calculated as unintentional injuries + the two intentional injury intents of homicide and suicide. The sum of costs across injury categories (excluding homicide and suicide) does not add up to the costs of unintentional injuries because categories include intentional and unintentional injuries

### Total costs of annual injury incidents and deaths

Findings suggest total costs of $4.5 billion, comprising the lifetime medical costs of annual incident injuries among IHS users and mortality costs of annual injury deaths among the AI/AN service population (Table 3). The highest-combined lifetime medical and mortality costs were for motor vehicle/traffic injuries ($1.6 billion), followed by poisonings ($960 million) and firearm-related injuries ($541 million).

**Table 3:** Lifetime medical and mortality costs of annual AI/AN injuries by injury intent and cause^a^

Injury category	Lifetime Medical Cost of Annual Injury Incidents (IHS users, 2017 USD thousands)	Mortality Cost (IHS service population, 2017 USD thousands)		Total Cost (2017 USD thousands)
		Labor Productivity Losses	Household Productivity Losses	
Costs by Injury Intent				
Unintentional	362,232	2,151,237	509,212	3,022,681
Self-inflicted/suicide	9127	645,864	139,807	794,798
Assault/homicide	38,125	377,968	78,407	494,500
Other	468	–	–	468
Undetermined	138,845	–	–	138,845
Total Costs	548,796	3,175,069	727,427	4,451,292
Costs by Injury Cause				
Motor vehicle/traffic	40,512	1,284,615	297,032	1,622,159
Poisoning	11,400	759,675	189,130	960,205
Firearm	1012	453,386	87,223	541,621
Pedestrian-related	744	233,027	50,830	284,601
Falls	130,593	63,824	14,549	208,967
Drowning	145	91,822	18,376	110,342
Fire/burn	6110	55,122	14,335	75,567

^a^**Note:** Table 3 shows estimated medical costs for treated injury events among 2011–2015 IHS users divided by five to reflect estimated annual non-fatal and fatal injury events. Estimated mortality costs, productivity losses for deaths among AI/AN in the 2008–2010 IHS service population are divided by three to reflect annual injury deaths. Costs assume that deaths with unintentional, suicide, and homicide as intents reflect all injury deaths. The Costs by Injury Cause section shows costs for selected causes that align closely with the cause of death categories. Some costs may be incurred in multiple categories. For example, the motor vehicle/traffic category includes pedestrian-related motor vehicle crashes and all other motor vehicle injuries

## Discussion

The estimated rates of injury found in this study were similar to rates found in previous studies, with a non-fatal injury incidence of 12,202 per 100,000 person years among IHS users versus 10,728 in 2000 (Piland, 2003). Although injury rates in this study were similar to those reported previously for falls, fires and burns, poisonings, firearms, and drownings (Piland, 2003), the estimated incidence of motor vehicle injuries was 527 per 100,000, representing a 57% reduction from the previously reported incidence rate of 1218 (Piland, 2003). Annual deaths due to unintentional injury among AI/ANs in the IHS service population increased to an estimated 1914, compared with 1353 annual deaths reported in 2000 (Piland, 2003), with poisoning as the largest contributor. The estimated 660 annual poisoning deaths from 2008–2010 is almost 3.5 times the 192 annual poisoning deaths reported in 2000 (Piland, 2003). The IHS Injury Report (Indian Health Service. Indian health focus, 2017) noted that while death rates for other injury categories have decreased over time, death rates from poisonings have increased significantly in recent decades, likely due to an increase in deaths from opioid overdoses (Chen et al., 2014).

This analysis showed that over half of the injuries among IHS users that were coded as self-inflicted had a reported cause of poisoning (Additional file 3 ). Another 22% had a cause of cut/pierce, and 5% had a cause of suffocation; 19% of self-inflicted injuries had a cause of “other.” Information about the causes of suicide deaths among AI/AN in the IHS service population was not available, but suicide rates in this population have exceeded rates for all races in the United States since at least the early 1970s (Indian Health Service. Indian health focus, 2017). Rates of suicide deaths in 2008–2010 were 19.6 per 100,000 for AI/AN in the IHS service population, which is 66% higher than the U.S. all races rate of 11.8 (Indian Health Service. Indian health focus, 2017).

In this study, productivity losses due to injury were valued using national wages as a proxy for employee output. Valuation of health using AI/AN-specific wages and employment rates, rather than national wages and employment rates, would result in a lower value, but assigning different monetary worth to the health of members from different race/ethnicity groups for decision-making raises issues of equity. Thus, the use of general wages and employment rates, rather than race-/ethnicity-specific wages and employment rates, is appropriate for this study.

Study findings suggest that past IHS investments in injury prevention may be paying off in terms of reduced injuries and medical costs among IHS users from 2011–2015 compared with 2000. For example, this study found a motor vehicle/traffic incidence of 527 per 100,000 IHS users, which is less than half the incidence (1218 per 100,000) reported in 2000 (Piland, 2003). Similarly, this study found motor vehicle/traffic-associated lifetime medical costs of $203 million (in 2017 USD) among IHS users from 2011–2015 (approximately $41 million per year). The previously reported estimate for motor vehicle/traffic injuries was seven times higher at $285 million per year (in 2000 USD) (Piland, 2003). Study findings suggest that prevention strategies likely contributed to the large reduction in motor vehicle/traffic injuries (Piland N, Berger L. The economic burden of injuries involving American Indians and Alaska Natives: a critical need for prevention. IHS Prim Care Provid [Internet], 2007; Billie et al., 2016). For example, the Tribal Motor Vehicle Injury Prevention Program was implemented in selected tribal communities between 2004 and 2013 and led to increased child safety seat use, reduced motor vehicle crashes with injuries or fatalities, and cost benefits.

The IHS 2018 Congressional Justification cites other AI/AN injury prevention successes. The justification describes that “the IHS Injury Prevention Program has been instrumental in reducing the injury mortality rate of AI/AN by 58% since it moved from an ‘education only’ focus to a public health approach” (U.S. Department of Health and Human Services, 2018). Additional injury prevention needs identified in the 2018 justification include prevention of unintentional injuries among AI/AN ages 1–44, suicide and violence prevention initiatives, and prescription drug overdose prevention activities. The high cost of these injuries for IHS, tribal programs, and other stakeholders that support the health-related needs of AI/AN justifies the focus on preventing them.

### Limitations

This analysis had several limitations. First, because the external cause of injury codes are not used for billing, coding can be inconsistent or not assigned (Hunt et al., 2018; Lawrence et al., 2006). As a result, 68,976 records in the 2011–2015 IHS user population data had missing or invalid external cause of injury codes and were not included in cost estimates for this study. Assigning an average cost of $978 per event for these injuries, the average cost across causes for injuries treated in a doctor’s office (Additional file 2 ) would add $67 million to injury cost estimates, raising the total cost by 2.4%.

Second, costs may be under-reported due to a lack of data on injury cause or setting. Additionally, cost estimates were not available for some settings (e.g., telemedicine). This cost analysis excluded 16% of injuries due to missing information, approximately 7% of which were missing an injury cause (Additional file 1).

Third, transportation costs may be undercounted because they do not take into account the potentially higher transportation costs incurred when injuries occur in or are initially treated in rural areas, such as on reservations or in IHS clinics in rural areas. For example, severe injuries occurring in rural areas may require transportation by medevac to a large hospital with a trauma unit.

Fourth, lifetime medical costs per injury encounter for doctor’s office and outpatient settings were based on estimates of medical spending from the late 1990s and early 2000s, using the 1996–1999 Medical Expenditure Panel Survey and 2000 Health Care Utilization Project—National Inpatient Sample. Because treatments may have changed, medical cost estimates may overstate or understate injury treatment costs.

Fifth, the analysis did not include several potential impacts of injury, such as productivity losses for non-fatal injuries or caregiving costs. Also excluded were quality of life losses resulting from injury and the value of those losses. Other informal health care sector costs excluded from the estimates were equipment purchases or home modification expenses for permanent injury-related disabilities. Additionally, AI/AN who resided in the IHS service population but were treated for injuries outside IHS were not captured in medical cost estimates. The IHS user population averaged 1.6 million people, and the service population averaged 2.3 million people. Thus, the analysis provides a conservative estimate of injury costs for the IHS service population.

Sixth, the data were from two different time periods. Data for health care use to treat injuries among IHS users were from 2011–2015, the most recent period available at the time of analysis. However, the most recently available data on injury deaths among AI/AN in the IHS service population were for 2008–2010. Because of the discrepancy in data periods, the death data may not fully reflect reductions in injury deaths resulting from recent IHS targeted investments, such as efforts targeting opioid misuse.

## Conclusions

The lifetime costs of annual injuries treated among IHS users and of annual injury deaths among AI/AN in the IHS service population likely exceed $4.4 billion. Given that the IHS user population averaged 1.6 million people during 2011–2015, these estimates suggest injury-related lifetime medical costs of more than $340 per IHS user per year. Additionally, injury deaths among the IHS service population of 2.3 million people for 2008–2010 suggest injury-related mortality costs of almost $1700 per AI/AN in the service population per year. Although these analyses used IHS data, they may have applicability for many of the 5.2 million people in the United States who identified as AI/AN in the 2010 Census (U.S. Census Bureau, 2010). Findings highlight a need for investments in injury prevention to reduce loss of life, loss of productive life before 75, medical costs, and productivity losses from injuries among AI/AN. Results also suggest potential target areas for future injury prevention, such as expanding efforts for fall prevention, poisoning treatment and prevention, addressing firearm-related injury, and continuing motor vehicle/traffic injury prevention. Further study of the factors contributing to high rates of each of these injury causes may be needed to best guide prevention efforts. Prior research found that alcohol contributed to a higher rate of mortality from poisoning, assault, drowning, fire injury, motor vehicle crashes, suicide, and falls among AI/AN compared with non-Hispanic Whites (Landen et al., 2014). Additionally, because 13.7% of injuries (136,359) from the NDW had an unspecified cause and an additional 68,976 had invalid injury cause codes, approaches to improve injury reporting among IHS providers should be a priority to allow for improved targeting of injury prevention efforts. Considering the high costs of injury treatment and large productivity losses, IHS investments in evidence-based injury prevention efforts may be more than offset by cost savings from reduced medical care and productivity losses. Finally, although these analyses used data on AI/AN in IHS service populations, the findings may also be useful for guiding policy more generally to address the underlying causes of injury among AI/AN in the United States.

## Supplementary information

**Additional file 1.** Identification of injuries and missing codes in NDW data.

**Additional file 2.** Published estimates of lifetime medical costs in 2017 USD.

**Additional file 3.** Non-fatal injury incidence by cause and intent among IHS users, 2011–2015.

## Data Availability

The datasets generated and/or analyzed during the current study are not publicly available due to a data use agreement specific to this study given that IHS is a health plan that limits the use and disposition of the data to the current project.

## Abbreviations

AI/AN: American Indians and Alaska Natives
BS/AO: Being struck by or against objects
CDC: Centers for Disease Control and Prevention
IHS: Indian Health Service
NCIPC: National Center for Injury Prevention and Control
NDW: National Data Warehouse
PRC: Purchased/referred care
USD: U.S. dollars
WISQARS: Web-based Injury Statistics Query and Reporting System
YPLL: Years of potential life lost

## Footnotes

1 The time period for the NDW data was October 1, 2010–September 30, 2015. Consequently, the injury coding in these data relies on ICD-9-CM codes, rather than ICD-10-CM codes, which were formally implemented on October 1, 2015.

2 Emergency department settings included discharged emergency department patients and those coded as “observation” with a length of stay of 1 day or shorter.

3 Transfer settings included Medivax transfers (*n* = 122), which made up less than 1% of all 16,277 transfers.

4 Hospitalization settings included injuries coded as “admission” and all non-fatal injuries with a stay longer than 1 day.
