# Supplementary material for: Incidence, deaths, and lifetime costs of injury among American Indians and Alaska Natives
Source: Inj Epidemiol. 2019 Nov 11;6:44. doi: 10.1186/s40621-019-0221-z (PMC6844062; doi:10.1186/s40621-019-0221-z)
Supplement: Supplementary file 1 — Additional file 1. Identification of injuries and missing codes in NDW data. [file 40621_2019_221_MOESM1_ESM.docx]

Appendix 1: Identification of injuries and missing codes in NDW data

This supplementary file provides details on the injury codes (E-codes) used to identify the specific causes and intents of each injury in the NDW data obtained for this analysis. It also describes the number of injury events excluded for different components of the analysis and discusses why they were excluded. The coding recommended by the CDC NCIPC for conducting injury analyses, as shown in Table A1, was used to identify injury causes and intents.

Table A1 Recommended injury codes and categories from the NCIPC

| Cause | Manner/Intent | | | | |
| --- | --- | --- | --- | --- | --- |
|  | Unintentional | Self-inflicted | Assault | Undetermined | Other |
| Cut/pierce | E920.0–.9 | E956 | E966 | E986 | E974, E995.2 |
| Drowning/submersion | E830.0–.9  E832.0–.9  E910.0–.9 | E954 | E964 | E984 | E995.4 |
| Fall | E880.0-E886.9  E888 | E957.0–.9 | E968.1 | E987.0–.9 |  |
| Fire/burn | E890.0-E899  E924.0–.9 | E958.1,.2,.7 | E961  E968.0,.3  E979.3 | E988.1,.2,.7 |  |
| Fire/flame | E890.0-E899 | E958.1 | E968.0  E979.3 | E988.1 |  |
| Hot object/substance | E924.0–.9 | E958.2,.7 | E961  E968.3 | E988.2,.7 |  |
| Firearm | E922.0–.3,.8, .9 | E955.0–.4 | E965.0–4  E979.4 | E985.0–.4 | E970 |
| Machinery | E919 (.0–.9) |  |  |  |  |
| Motor vehicle traffic | E810-E819 (.0–.9) | E958.5 | E968.5 | E988.5 |  |
| Occupant | E810-E819 (.0,.1) |  |  |  |  |
| Motorcyclist | E810-E819 (.2,.3) |  |  |  |  |
| Pedal cyclist | E810-E819 (.6) |  |  |  |  |
| Pedestrian | E810-E819 (.7) |  |  |  |  |
| Unspecified | E810-E819 (.9) |  |  |  |  |
| Pedal cyclist, other | E800-E807 (.3) |  |  |  |  |
|  | E820-E825 (.6)  E826.1,.9 |  |  |  |  |
|  | E827-E829(.1) |  |  |  |  |
| Pedestrian, other | E800–807(.2) |  |  |  |  |
|  | E820-E825(.7) |  |  |  |  |
|  | E826-E829(.0) |  |  |  |  |
| Transport, other | E800-E807 (.0,.1,.8,.9) | E958.6 |  | E988.6 |  |
|  | E820-E825 (.0–.5,.8,.9) |  |  |  |  |
|  | E826.2–.8 |  |  |  |  |
|  | E827-E829 (.2–.9) |  |  |  |  |
|  | E831.0–.9  E833.0-E845.9 |  |  |  |  |
| Natural/environmental | E900.0-E909  E928.0–.2 | E958.3 |  | E988.3 |  |
| Bites and stings | E905.0–.6,.9  E906.0–.4,.5,.9 |  |  |  |  |
| Overexertion | E927.0–.4,.8–.9 |  |  |  |  |
| Poisoning | E850.0-E869.9 | E950.0-E952.9 | E962.0–.9  E979.6,.7 | E980.0-E982.9 | E972 |
| Struck by, against | E916-E917.9 |  | E960.0; E968.2 |  | E973  E975  E995 (.0,.1) |
| Suffocation | E911-E913.9 | E953.0–.9 | E963 | E983.0–.9 | E995.3 |
| Other specified and classifiable | E846-E848, E914-E915 | E955.5,.6,.7,.9 | E960.1  E965.5–.9 | E985.5,.6,.7 | E971  E978, |
|  | E918  E921.0–.9  E922.4,.5 | E958.0,.4 | E967.0–.9, | E988.0,.4 | E990-E994  E996 |
|  | E923.0–.9  E925.0-E926.9 |  | E968.4,.6, .7 |  | E997.0–.2 |
|  | E928(.3–.7)  E929.0–.5 |  | E979 (.0–.2,.5,.8,.9) |  |  |
| Other specified, not elsewhere classifiable | E928.8  E929.8 | E958.8  E959 | E968.8  E969  E999.1 | E988.8  E989 | E977  E995 (.8,.9  E997.8 |
|  |  |  |  |  | E998  E999.0 |
| Unspecified | E887  E928.9  E929.9 | E958.9 | E968.9 | E988.9 | E976  E997.9 |

Injuries with a Missing Injury Setting or Invalid External Cause of Injury Code

Some injuries were excluded from incidence counts, rates, and costs because they had a missing setting (*n* = 20,061) or an invalid external cause of injury code (*n* = 68,976). Invalid external cause of injury codes were either (1) codes that were not included in the NCIPC’s list of non-fatal injury codes or (2) place of occurrence injury codes that were mistakenly listed first (E849.XX) for the encounter. The study team attempted to assign causes to the 68,976 unassigned injury encounters by checking second- and third-listed codes and code descriptions for extra information. However, these additional injuries represented a very small number of injuries relative to the total number of injuries with causes/settings assigned (2700, or 0.27%). The study team therefore left these injuries out of calculations of lifetime medical costs.

Furthermore, some injuries had settings coded as “chart review” or “telemedicine” (*n* = 1670). These injuries represented a very small proportion of total injuries (~ 0.1%), and the cost of these services was likely to be minimal compared with the cost of higher-intensity medical services. The study team therefore excluded all injuries with settings of chart review or telemedicine from the analysis.

Finally, some setting-cause combinations had no associated costs to assign from the published injury cost estimates in Finkelstein et al. (2006) or WISQARS (Lawrence & Miller, 2014; *n* = 81,460). These combinations are represented by blanks in Appendix Table 5. The 349 fatal injuries included in the IHS sample were assigned medical costs using the unit cost estimates by sex and cause for fatal injuries from WISQARS (Lawrence & Miller, 2014). Overall, 914,014 out of 1,086,530 injury encounters (84%) in the NDW data were assigned medical costs. Figure A1 illustrates each of the exclusions used for analysis and the resulting sample.


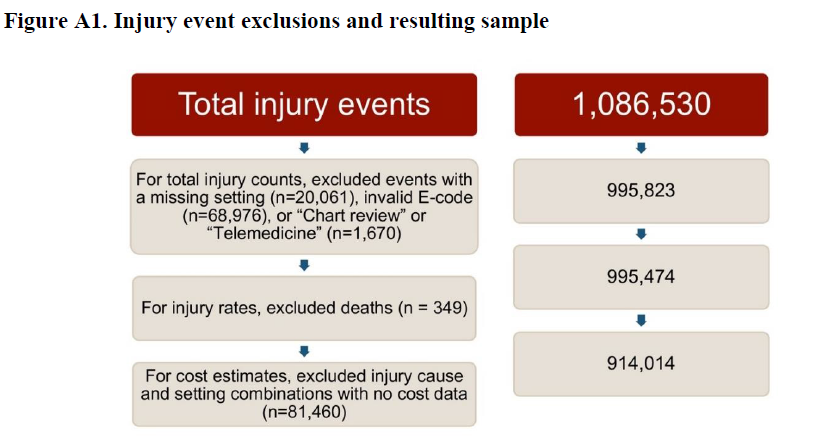


Note: Using the 2011-2015 NDW data extract, we identified 1,086,530 injury events. The figure shows how many remained after excluding those that could not be coded with cause or intent, resulted in death, and for which costs could not be assigned.

A sensitivity analysis was conducted to examine the likely impact on total 2011–2015 medical costs of including costs for the 20,061 injuries with missing setting information, the 68,976 injuries with invalid E-codes, and the 81,460 injuries with missing cost information. Applying a conservative cost estimate of $978 per injury event in 2017 USD, the average cost of the 170,497 injuries treated in doctors’ offices across all causes and both sexes would be $166.7 million, increasing the total 2011–2015 medical cost to $2,911 million, or by 6%.
