# Supplementary material for: Incidence, deaths, and lifetime costs of injury among American Indians and Alaska Natives
Source: Inj Epidemiol. 2019 Nov 11;6:44. doi: 10.1186/s40621-019-0221-z (PMC6844062; doi:10.1186/s40621-019-0221-z)
Supplement: Supplementary file 2 — Additional file 2. Published estimates of lifetime medical costs in 2017 USD. [file 40621_2019_221_MOESM2_ESM.docx]

Appendix 2. Published estimates of lifetime medical costs in 2017 USD

Table B1 shows estimates of lifetime medical costs by injury cause and care setting used in the study. These medical cost estimates were obtained from the published literature for all races/ethnicities in the U.S. population and inflated to 2017 USD using the Personal Consumption Expenditures for Health index. They reflect the present value of lifetime medical costs for each category, assuming a 3% annual discount rate. Cost estimates were applied to the counts of injuries by cause and setting from the NDW extract to estimate injury costs for 2011–2015 for the IHS user population. On average, 77% of lifetime medical costs per incident injury were incurred in Months 0–18 following the injury (Lawrence & Miller, 2014).

Table B1 Lifetime medical costs per injury incident by sex, cause, and setting (all races/ethnicities)^a^

| Cause | Costs by Setting (inflated to 2017 USD) | | | | | | | |
| --- | --- | --- | --- | --- | --- | --- | --- | --- |
|  | Doctor’s Office | Out-patient | Emergency Department | | Hospitalized | | Fatal | |
|  |  |  | WISQARS | Finkel-stein | WISQARS | Finkel-stein | WISQARS | Finkel-stein |
| Males | | | | | | | | |
| MV occupant | 2660 | 1032 | 3752 | 2057 | 65,411 | 49,203 | 10,194 | 10,402 |
| Motorcyclist | 890 | 1607 | 3709 | 1983 | 62,148 | 54,641 | 14,443 | 13,504 |
| Pedal-cyclist | 1608 | 1625 | 3299 | 1781 | 60,389 | 33,525 | 18,967 | 19,214 |
| Pedestrian | – | 1802 | 3598 | 1963 | 70,115 | 63,564 | 14,185 | 14,146 |
| Motor vehicle traffic, unspecified | 1425 | 1589 | 3653 | – | 63,779 | 42,021 | 11,852 | 9828 |
| Other transport | 1551 | 1558 | 2794 | 1983 | 47,692 | 36,469 | 5734 | 7118 |
| Fall | 1351 | 1501 | 2771 | 1797 | 48,588 | 30,861 | 24,809 | 24,119 |
| Struck by/against | 1242 | 1519 | 2392 | 1586 | 36,876 | 27,240 | 15,104 | 13,274 |
| Machinery | 1057 | 936 | 2070 | 1690 | 27,649 | 24,729 | 8710 | 12,403 |
| Firearm/gunshot | 500 | 1333 | 2870 | 1288 | 24,529 | 55,287 | 6639 | 4544 |
| Cut/pierce | 723 | 1307 | 1818 | 1294 | 17,576 | 16,968 | 12,268 | 7804 |
| Poisoning | 537 | 455 | 1855 | 1749 | 14,173 | 10,481 | 4967 | 4698 |
| Fire/burn | 340 | 1357 | 1884 | 2411 | 31,554 | 29,756 | 15,919 | 24,258 |
| Inhalation/suffocation | 166 | 393 | 1761 | 849 | 73,399 | 39,069 | 10,517 | 7890 |
| Drown/submersion | – | – | 1976 | 2435 | 38,402 | 33,996 | 6437 | 4477 |
| Bite/sting | 556 | 1297 | 2045 | 1167 | 20,048 | 10,154 | – | 14,732 |
| Natural/environ-mental | 1259 | 1282 | 2343 | 2385 | 39,855 | 26,162 | 10,812 | 9129 |
| Overexertion | 1100 | 1391 | 2788 | 2166 | 35,203 | 18,682 | 11,332 | 10,440 |
| Other specified | 854 | 964 | 1863 | 1560 | 16,215 | 27,123 | 18,443 | 15,609 |
| Unspecified | – | – | 2820 | 2024 | 41,478 | 27,897 | 31,390 | 18,517 |
| Females | | | | | | | | |
| MV occupant | 1320 | 1106 | 3477 | 1693 | 54,900 | 37,881 | 11,274 | 11,968 |
| Motorcyclist | – | 1372 | 3523 | 2069 | 57,490 | 50,642 | 17,119 | 16,462 |
| Pedal-cyclist | 983 | 1140 | 3415 | 1693 | 53,611 | 23,818 | 13,719 | 22,022 |
| Pedestrian | 957 | 1156 | 3401 | 1705 | 63,378 | 47,051 | 15,832 | 15,939 |
| Motor Vehicle Traffic, unspecified | 1327 | 1502 | 3446 | – | 56,195 | 31,901 | 12,581 | 10,605 |
| Other transport | 1196 | 1410 | 2635 | 1771 | 46,880 | 34,935 | 7500 | 11,761 |
| Fall | 1456 | 1462 | 2911 | 1623 | 40,093 | 24,790 | 27,253 | 25,875 |
| Struck by/against | 822 | 1380 | 2409 | 1546 | 32,256 | 21,582 | 19,379 | 15,187 |
| Machinery | 564 | 1082 | 1959 | 1480 | 29,727 | 22,541 | 11,850 | 7202 |
| Firearm/gunshot | – | 908 | 2841 | 1348 | 25,963 | 57,820 | 6139 | 3950 |
| Cut/pierce | 653 | 1395 | 1838 | 1300 | 12,087 | 12,163 | 5604 | 5576 |
| Poisoning | 576 | 443 | 2209 | 1632 | 13,072 | 9178 | 6274 | 6938 |
| Fire/burn | 393 | 1090 | 2027 | 2406 | 35,262 | 26,347 | 14,386 | 27,065 |
| Inhalation/suffocation | 961 | 385 | 1889 | 896 | 67,056 | 36,492 | 16,999 | 13,866 |
| Drown/submersion | – | – | 1644 | 2113 | 37,650 | 39,035 | 9812 | 6112 |
| Bite/sting | 540 | 1273 | 2200 | 1125 | 19,789 | 9746 | – | 18,377 |
| Natural/environ-mental | 1026 | 704 | 2362 | 2276 | 52,137 | 24,273 | 11,131 | 12,681 |
| Overexertion | 963 | 1447 | 2659 | 2585 | 36,593 | 18,828 | 3002 | 11,811 |
| Other specified | 792 | 992 | 2013 | 1468 | 14,220 | 24,125 | 23,673 | 17,938 |
| Unspecified | – | – | 2815 | 1114 | 38,833 | 22,442 | 40,537 | 21,897 |

**^a^Note:** Costs were obtained from 2010 WISQARS Data (16) and Finkelstein et al. (17). WISQARS costs (Lawrence & Miller, 2014) were used for emergency department-treated, hospitalized, and fatal injuries. Doctor’s office and outpatient injury costs were taken from Finkelstein et al. (2006). Blanks indicate that the source listed did not provide a cost estimate for the cause and setting combination.
