# Supplementary material for: Incidence, deaths, and lifetime costs of injury among American Indians and Alaska Natives
Source: Inj Epidemiol. 2019 Nov 11;6:44. doi: 10.1186/s40621-019-0221-z (PMC6844062; doi:10.1186/s40621-019-0221-z)
Supplement: Supplementary file 3 — Additional file 3. Non-fatal injury incidence by cause and intent among IHS users, 2011–2015. [file 40621_2019_221_MOESM3_ESM.docx]

Appendix 3. Non-fatal injury incidence by cause and intent among IHS users, 2011–2015

Table C1 shows the frequency of injuries by cause (i.e., mechanism) and intent (i.e., manner). The estimates are for non-fatal injuries only, although the IHS data included a limited subset of fatal injuries, which are included in Table 1. The fatal injuries in the NDW data represent fatalities treated in IHS clinics or hospitals. For each non-fatal injury cause and intent shown in Table C1, the following statistics are provided from the 2011–2015 data: (1) number of injuries with each cause/intent, (2) the percentage of total injuries with the given cause and intent, (3) the percentage of injuries of a given cause (e.g., out of all motor vehicle traffic injuries) with each intent, and (4) the percentage of injuries of a given intent (e.g., unintentional) with the given cause. For example, for firearms, the estimates in Table C1 indicate that 908, or < 0.1% of all injuries, were unintentional firearm injuries. For firearm injuries, 60% were unintentional, 9% were self-inflicted, 18% were assaults, and 11% were of other or undetermined intent. Similarly, out of all unintentional injuries (*n* = 868,589), 5% had a motor vehicle traffic-related cause.

Table C1 Non-Fatal Injuries (by Cause and Intent) among AI/AN in the IHS User Population, 2011–2015

| Injury cause | Injury Intent | | | | | | |
| --- | --- | --- | --- | --- | --- | --- | --- |
|  | Number/Percent | Unintentional | Self–Inflicted | Assault | Other | Undetermined | Total |
| Motor vehicle traffic | Number | 42,600 | 35 | 138 | 0 | 231 | 43,004 |
|  | % of all injuries with MVT cause and specified intent | 4.28 | 0.00 | 0.01 | 0.00 | 0.02 | 4.32 |
|  | % of MVT cause with specified intent | 99.06 | 0.08 | 0.32 | 0.00 | 0.54 | 100.00 |
|  | % of specified intent with MVT cause* | 4.96 | 0.31 | 0.16 | 0.00 | 0.59 | 4.32 |
| Firearm | Number | 819 | 126 | 231 | 47 | 149 | 1372 |
|  | % of total with specified intent | 0.08 | 0.01 | 0.02 | 0.00 | 0.01 | 0.14 |
|  | % of cause with intent | 59.69 | 9.18 | 16.84 | 3.43 | 10.86 | 100.00 |
|  | % of intent with cause | 0.10 | 1.13 | 0.27 | 3.41 | 0.38 | 0.14 |
| Poisoning | Number | 8115 | 5615 | 85 | 11 | 3020 | 16,846 |
|  | % of total with specified intent | 0.82 | 0.56 | 0.01 | 0.00 | 0.30 | 1.69 |
|  | % of cause with intent | 48.17 | 33.33 | 0.50 | 0.07 | 17.93 | 100.00 |
|  | % of intent with cause | 0.95 | 50.31 | 0.10 | 0.80 | 7.70 | 1.69 |
| Falls | Number | 236,082 | 74 | 118 | 0 | 674 | 236,948 |
|  | % of total with specified intent | 23.72 | 0.01 | 0.01 | 0.00 | 0.07 | 23.80 |
|  | % of cause with intent | 99.63 | 0.03 | 0.05 | 0.00 | 0.28 | 100.00 |
|  | % of intent with cause | 27.49 | 0.66 | 0.14 | 0.00 | 1.72 | 23.80 |
| Suffocation | Number | 1054 | 589 | 143 | 0 | 41 | 1827 |
|  | % of total with specified intent | 0.11 | 0.06 | 0.01 | 0.00 | 0.00 | 0.18 |
|  | % of cause with intent | 57.69 | 32.24 | 7.83 | 0.00 | 2.24 | 100.00 |
|  | % of intent with cause | 0.12 | 5.28 | 0.17 | 0.00 | 0.10 | 0.18 |
| Drowning | Number | 210 | 5 | 2 | 0 | 1 | 218 |
|  | % of total with specified intent | 0.02 | 0.00 | 0.00 | 0.00 | 0.00 | 0.02 |
|  | % of cause with intent | 96.33 | 2.29 | 0.92 | 0.00 | 0.46 | 100.00 |
|  | % of intent with cause | 0.02 | 0.04 | 0.00 | 0.00 | 0.00 | 0.02 |
| Fire/burn | Number | 20,120 | 86 | 112 | 0 | 347 | 20,665 |
|  | % of total with specified intent | 2.02 | 0.01 | 0.01 | 0.00 | 0.03 | 2.08 |
|  | % of cause with intent | 97.36 | 0.42 | 0.54 | 0.00 | 1.68 | 100.00 |
|  | % of intent with cause | 2.34 | 0.77 | 0.13 | 0.00 | 0.88 | 2.08 |
| Cut/pierce | Number | 65,718 | 2493 | 4044 | 42 | 498 | 72,795 |
|  | % of total with specified intent | 6.60 | 0.25 | 0.41 | 0.00 | 0.05 | 7.31 |
|  | % of cause with intent | 90.28 | 3.42 | 5.56 | 0.06 | 0.68 | 100.00 |
|  | % of intent with cause | 7.65 | 22.34 | 4.76 | 3.05 | 1.27 | 7.31 |
| Struck by/against | Number | 109,874 | 0 | 44,789 | 975 | 0 | 155,638 |
|  | % of total with specified intent | 11.04 | 0.00 | 4.50 | 0.10 | 0.00 | 15.63 |
|  | % of cause with intent | 70.60 | 0.00 | 28.78 | 0.63 | 0.00 | 100.00 |
|  | % of intent with cause | 12.80 | 0.00 | 52.67 | 70.81 | 0.00 | 15.63 |
| Machinery | Number | 3315 | 0 | 0 | 0 | 0 | 3315 |
|  | % of total with specified intent | 0.33 | 0.00 | 0.00 | 0.00 | 0.00 | 0.33 |
|  | % of cause with intent | 100.00 | 0.00 | 0.00 | 0.00 | 0.00 | 100.00 |
|  | % of intent with cause | 0.39 | 0.00 | 0.00 | 0.00 | 0.00 | 0.33 |
| Other pedal cyclist | Number | 8013 | 0 | 0 | 0 | 0 | 8013 |
|  | % of total with specified intent | 0.80 | 0.00 | 0.00 | 0.00 | 0.00 | 0.80 |
|  | % of cause with intent | 100.00 | 0.00 | 0.00 | 0.00 | 0.00 | 100.00 |
|  | % of intent with cause | 0.93 | 0.00 | 0.00 | 0.00 | 0.00 | 0.80 |
| Other pedestrian | Number | 1247 | 0 | 0 | 0 | 0 | 1247 |
|  | % of total with specified intent | 0.13 | 0.00 | 0.00 | 0.00 | 0.00 | 0.13 |
|  | % of cause with intent | 100.00 | 0.00 | 0.00 | 0.00 | 0.00 | 100.00 |
|  | % of intent with cause | 0.15 | 0.00 | 0.00 | 0.00 | 0.00 | 0.13 |
| Other transport | Number | 21,291 | 0 | 0 | 0 | 1 | 21,292 |
|  | % of total with specified intent | 2.14 | 0.00 | 0.00 | 0.00 | 0.00 | 2.14 |
|  | % of cause with intent | 100.00 | 0.00 | 0.00 | 0.00 | 0.00 | 100.00 |
|  | % of intent with cause | 2.48 | 0.00 | 0.00 | 0.00 | 0.00 | 2.14 |
| Natural/environmental | Number | 84,956 | 5 | 0 | 0 | 149 | 85,110 |
|  | % of total with specified intent | 8.53 | 0.00 | 0.00 | 0.00 | 0.01 | 8.55 |
|  | % of cause with intent | 99.82 | 0.01 | 0.00 | 0.00 | 0.18 | 100.00 |
|  | % of intent with cause | 9.89 | 0.04 | 0.00 | 0.00 | 0.38 | 8.55 |
| Overexertion | Number | 97,766 | 0 | 0 | 0 | 0 | 97,766 |
|  | % of total with specified intent | 9.82 | 0.00 | 0.00 | 0.00 | 0.00 | 9.82 |
|  | % of cause with intent | 100.00 | 0.00 | 0.00 | 0.00 | 0.00 | 100.00 |
|  | % of intent with cause | 11.39 | 0.00 | 0.00 | 0.00 | 0.00 | 9.82 |
| Other | Number | 157,493 | 2132 | 35,379 | 302 | 34,111 | 229,417 |
|  | % of total with specified intent | 15.82 | 0.22 | 3.56 | 0.03 | 3.43 | 23.05 |
|  | % of cause with intent | 68.65 | 0.93 | 15.42 | 0.13 | 14.87 | 100.00 |
|  | % of intent with cause | 18.35 | 19.10 | 41.60 | 21.93 | 86.97 | 23.05 |
| Total | Number | 858,673 | 11,160 | 85,041 | 1377 | 39,222 | 995,473 |
|  | % of total with specified intent | 86.26 | 1.12 | 8.54 | 0.14 | 3.94 | 100.00 |
|  | % of cause with intent | 86.26 | 1.12 | 8.54 | 0.14 | 3.94 | 100.00 |
|  | % of intent with cause | 100.00 | 100.00 | 100.00 | 100.00 | 100.00 | 100.00 |
